# Graduate nurses caring for people living with dementia: A scoping review

**DOI:** 10.1177/14713012251313489

**Published:** 2025-01-06

**Authors:** Jerric Jose, Elisabeth Jacob, Louise Alexander

**Affiliations:** 95359 Monash Health & Australian Catholic University, Australia; 95359 Australian Catholic University, Australia; 2104 Deakin University, Australia

**Keywords:** graduate, nurse, dementia, Alzheimer, new

## Abstract

Background: Adequate dementia care knowledge of graduate nurses is essential for the wellbeing of both people living with dementia as well as the graduate nurses caring for them. Little is known about the dementia care knowledge, experience or confidence of graduate nurses caring for people living with dementia. This paper aims to review the available literature on graduate nurses caring for people living with dementia. Design: A scoping review of literature based on the Joanna Briggs Institute methodology for scoping reviews. Methods: From April 2023 to August 2023, searches were conducted in databases which included CINAHL, Medline, Embase, Web of Science, Scopus, Trove and Google search. All articles related to the question “what literature is available on graduate nurses caring for people living with dementia” published in English were included in this scoping review.

**Key findings**: Six articles were found that met our inclusion criteria. Thematic analysis identified three themes from the literature: (1) preparation to care for people living with dementia, (2) impact of dementia care training and (3) experience of caring of people living with dementia.

## Background

The World Health Organization (WHO) (2020) describes dementia as a syndrome of cognitive impairment that affects memory, behaviour and cognitive abilities, interfering significantly with a person’s ability to perform daily activities. The global aging population is increasing and as a consequence, the number of older multi-morbid and frail patients treated in general hospitals rises (Reynish et al., 2017). As the number of people living with dementia increases, there is a proportionate increase in the number of hospital presentation of people living with dementia (Australian Institute of Health and Welfare (AIHW), 2023; Williamson et al., 2021; Yorganci et al., 2022). This has led to a situation where the care of people living with dementia has become a key priority for health care organisations across the globe (WHO, 2020). Hospital presentations are more frequent among people living with dementia and it increases towards the end of their life (Williamson et al., 2021). People living with dementia are more likely to have longer stays and poorer outcomes in hospitals compared to general population (Alzheimer’s Association, 2024; Australian Commission on Safety and Quality in Health Care, 2016; Canadian Nurses Association (CAN), 2016).

Caring for people living with dementia in hospitals can be challenging due to a combination of pre-existing comorbidities, as well as cognitive and functional decline (Bail et al., 2013). Hospital admission can be frightening and confusing for people living with dementia, with hospital stays linked to physical and psychological deconditioning (Dewing & Dijk, 2016; Sampson et al., 2014). Hospitalisation can increase the behavioural and psychological symptoms of people living with dementia, increasing the risk of adverse effects and poor outcomes (Dewing & Dijk, 2016; Hermann et al., 2015; Sampson et al., 2014). During hospital admission, the risk of experiencing an adverse outcome is higher in people living with dementia compared to people without any cognitive impairment (Fogg et al., 2018). The most common adverse outcomes that may occur during hospitalisation of people living with dementia are hospital acquired pneumonia, delirium, urinary tract infection, faecal incontinence, gastroenteritis, adverse drug reactions, pressure ulcers and falls (Fogg et al., 2018). These adverse outcomes often cause functional decline, increased care needs and extended hospitalisation (Fogg et al., 2018; Reynish et al., 2017).

National Dementia Strategies in the United Kingdom have highlighted the significance of identifying and addressing the gaps in the dementia care knowledge and skills of nurses caring for people living with dementia (Department of Health, Social Services and Public Safety, 2011). Dementia-specific training programs have improved the knowledge, confidence and attitudes of nurses providing care for people living with dementia (Elvish et al., 2014; Jack-Waugh et al., 2018; Matsuda et al., 2018). Thus, dementia care training of nursing staff is a key factor in improving the quality of care and outcome of the people living with dementia.

Graduate nurses contribute a major portion of the nursing workforce across the globe (The Organization for Economic Cooperation and Development (OECD), 2021); Ward (2021). With an ongoing increase in the number of people living with dementia admitted to hospitals, graduate nurses are in a position to provide care for people living with dementia more frequently. But some universities lack sufficient focus on dementia care education in their undergraduate nursing curriculum (Baillie et al., 2015; Eccleston et al., 2015; Gould et al., 2015; Poreddi et al., 2015), limiting the dementia care knowledge of newly graduated nurses. Inadequate dementia care knowledge of nurses may affect their confidence, and the quality of care provided to people living with dementia (Yaghmour, 2022) and this may affect job satisfaction, increase stress and the risk of burn-out in the workplace (Rose et al., 2010; Smythe et al., 2020).

To ensure the provision of quality care by graduate nurses to people living with dementia, it is essential to identify the dementia care knowledge and skills of graduate nurses on commencement of their graduate programme, so upskilling programs can be developed. Although studies have identified the need for improved knowledge and skills in relation to dementia care (Chater & Hughes, 2013; Evripidou et al., 2019; Kada et al., 2009; Kang et al., 2017; Matsuda et al., 2018), there are currently no studies that have assessed the dementia care knowledge, confidence and experience of graduate nurses caring for people living with dementia. This situation highlights the need to review available literature on graduate nurses caring for people living with dementia.

## Aim

The aim of this scoping review is to review the available literature on graduate nurses caring for people living with dementia.

## Methods

This review followed the Joanna Briggs Institute methodology for scoping reviews (Peters et al., 2020). Reporting of the evidence was based on “Preferred Reporting Items for Systematic reviews and Meta-Analyses extension for Scoping Reviews (PRISMA-ScR) checklist” (Peters et al., 2021).

### Data sources and search strategy

Literature searches using five databases (CINAHL, Medline, Embase, Web of Science and Scopus), Trove and Google were conducted from April 2023 to August 2023. A health science librarian who is skilled in systematic review searches was consulted to assist in the creation of the search strategy. The full search was undertaken with key words, including dementia and graduate nurse, in both the titles and abstracts of the articles (Table 1).

**Table 1.** Search terms.

	Concept 1	Concept 2
Keywords (title & abstract) for all databases	(Graduate* OR “New* qualif*) AND nurse*	Dementia* OR alzheimer*
CINAHL (Headings)	MH New graduate nurses	Dementia
Medline (MeSH)	N/A	Dementia
Embase (Emtree)	N/A	Dementia
Web of science	N/A	N/A

### Eligibility criteria

All available evidence about graduate nurses involved in care of patients with dementia were considered for this study. Any studies, programmes or presentations exploring graduate nurses and dementia care were evaluated. There were no restrictions placed on publication dates, and only full text articles published in English were included.

### Screening process

**Figure 1.**
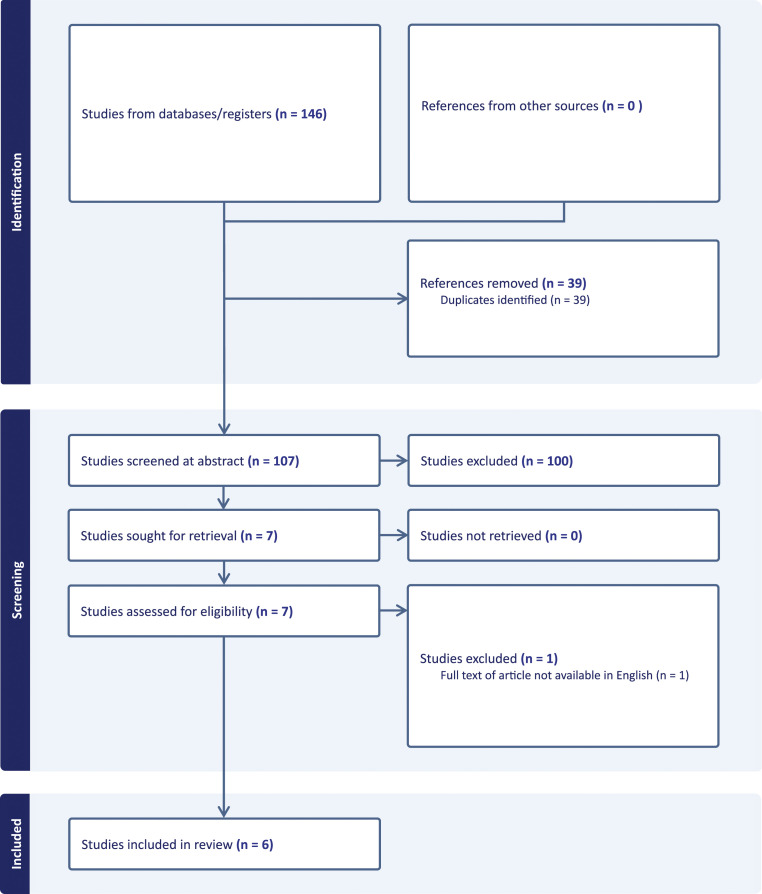
PRISMA flow chart.

The lead author with two co-authors completed the initial screening of title and abstracts to determine eligibility using Covidence (Babineau, 2014). The search initially produced 146 articles. After removing duplicates, 107 articles remained. Initial screening of titles and abstracts excluded 100 articles as these did not meet inclusion criteria. Conflicts were resolved in face-to-face meetings. Full text review was then undertaken of potential papers in strict compliance with inclusion and exclusion criteria. Full-text screening of the remaining seven articles yielded six articles which were eligible for inclusion in the review (Figure 1).

### Data extraction and synthesis of results

Data extraction was undertaken with the intent to identify content relevant to graduate nurses caring for patients with dementia. Characteristics of the selected article’s include author, year, country, aim/purpose/objective, setting/context, study population, study period, study design and key findings (Table 2). The lead author and the two reviewers (EJ, LA) independently extracted data from all the studies, and discussed the results. The extraction process involved several meetings and video conferences to refine the table contents. We then analyzed possible themes for each study which required a thorough review of what was being measured and the explicit rationale. This process required several article readings and discussions on identifying common themes from the articles.

**Table 2.** Characteristics of selected articles.

Author & year	Objectives/Aims/Purpose	Setting/context	Study population	Study period	Study design	Key findings
Sorrell & Szweda (2015)	To implement a simulated relationship-building experience that engaged nurse residents in a docudrama of families caring for older adults with dementia.	Cleveland Clinic, USA	New graduate nurses	Not specified	Qualitative study	The docudrama helped nurse residents gain empathy and understanding through stepping into the lives of families experiencing dementia.
Newman (2020)	Supporting the frontline workforce during COVID-19	East Metropolitan health Service, Australia	Newly qualified registered nurses	Not specified	Case report	All participants strongly agreed that the training had provided new knowledge or information about how to care for people living with dementia and all said their confidence in caring for a person with dementia had increased. All participants said that, as a result of the training, they would support someone else to change the way they care for or support a person with dementia; that they would try a new procedure or technique shared during the training; and would do further education or training.
Hebditch et al. (2022)	Preferences of newly qualified healthcare professionals for working with people with dementia: a Qualitative study	UK	Nurses and doctors within two years of qualification	Not specified	Qualitative study with semi-structured interviews	The results present six main categories representing complex interlinked factors influencing preferences for working with people with dementia as well as exploring the definition of a career working with people with dementia. The factors include the importance of making a difference; seeing dementia care as a different type of care; its perceived alignment with personal characteristics; perceptions of people with dementia; care environments and career characteristics.
Hartung et al. (2021)	New graduate nurses and dementia care in acute care: A qualitative study	Canada	New graduate nurses	July 2016 and January 2017	Qualitative study with semi-structured interviews	Three themes emerged from the thematic analysis: (1) building of vision and values; (2) clashing of vision and values; and (3) making do with what you have. Barriers to providing dementia care in acute care were similar to barriers experienced by non-new graduate nurses reported in the literature, such as challenges with responsive behaviours, maintaining safety and providing psychosocial care. Facilitators identified were supportive colleagues and early exposure to dementia care.
Dahlke et al. (2021)	Registered nurse’s reflections on their educational preparation to work with older people	Alberta health services, Canada	Recently graduated registered nurses	Not specified	Qualitative descriptive study	Nurses did not recognise the importance of learning about older people until they had graduated. Only then did they realise that the ageing population was so complex and prevalent. They perceived a lack of education particularly related to working with older people with dementia and their behaviours, as well as learning how to communicate to an older population. Participants perceived that as students, it was up to them to fit in learning about working with older people without the support of faculty.
Buenconsejo (2022)	Phenomenological study of the lived experience of New graduate nurses caring for hospitalized patients living with dementia	An acute care hospital in southern California, USA	New graduate nurses	Not specified	Qualitative study with semi-structured interviews	The thematic analysis yielded nine overall themes addressing two lines of inquiry. The themes discovered in the first line of inquiry included protecting patient’s universal rights, ensuring patient safety and well-being, complex care delivery experience, fostering therapeutic nurse-patient relationship, nurse’s positive adaptation and role transition. In the second line of inquiry, the themes included preservation of human connections, feeling inadequate and experiencing personal distress. The themes were analyzed over time and articulated into a cogent phenomenological lived experience.

## Results

Six articles were identified that mentioned graduate nurses caring for people living with dementia. All the six articles were qualitative studies (Buenconsejo, 2022; Dahlke et al., 2021; Hartung et al., 2021; Hebditch et al., 2022; Newman, 2020; Sorrell & Szweda, 2015). Studies were undertaken in Australia, Canada, United Kingdom (UK) and United States of America (USA).

### Themes

Three themes were identified during the review: (1) preparation to care for people living with dementia, (2) impact of dementia care training, and (3) experience of caring of people living with dementia.

#### Preparation to care for people living with dementia

This theme refers to the need for nurses to be adequately prepared to care for people living with dementia. Three studies, Dahlke et al. (2021), Newman (2020) and Hebditch et al. (2022) reported on the need for graduate nurses to be prepared to care for people living with dementia before they start working in clinical or non-clinical settings. All three studies agreed that graduate nurses have little confidence in caring for people with dementia due to lack of knowledge and experience (Dahlke et al., 2021; Hebditch et al., 2022; Newman, 2020). Similarly, (Dahlke et al. (2021), Newman (2020) and Hebditch et al. (2022) reported that caring for people living with dementia should be a part of the undergraduate curriculum. Including dementia care in the curriculum would assist with dispelling negative stereotypes about ageing, and facilitating interest in older people (Dahlke et al., 2021), prevent graduates being thrown in the deep end and required to care for patients without an understanding of their conditions (Newman, 2020), improve the confidence and competency of graduates to meet patient needs (Hebditch et al., 2022), and addressing skill deficits in caring for people living with dementia, such as communication and tolerance of ambiguity (Hebditch et al., 2022).

#### Impact of dementia care training

Newman (2020) conducted a training for newly graduated nurses aligning the fundamentals with the actual activities happening in the hospitals to support people living with dementia. Newman (2020) has reported that all participants who attended the training strongly agreed that the training had provided has improved their knowledge and confidence in caring for people living with dementia. The participants were confident enough to support their fellow nurses at their workplace to change the way they care for people living with dementia. They were determined to do further education and training on dementia care. The participants have suggested that universities should provide similar dementia care training before first practical placement.

Sorrell and Szweda (2015) conducted a docudrama that helped nurse graduates to gain empathy and understanding through stepping into the lives of families experiencing dementia. The docudrama has also helped them to gain insight into what it is like to be a caregiver for someone living with dementia. Nurse graduates mentioned that the docudrama encouraged them to identify the needs of people living with dementia and care for them in a way that honours each individual. Through docudrama, the graduate nurses identified that they could do more to ensure that the quality of life of people living with dementia was enhanced. They noted the need to be involved as much as possible in their patient’s plan of care and to recognize their patient’s preferences, likes, and dislikes, and incorporate those into their nursing care.

#### Experience of caring of people living with dementia

This theme refers to the experiences that graduate nurse’s encounters when caring for people living with dementia. Two papers, Hartung et al. (2021) and Buenconsejo (2022), described the experiences of graduate nurses in caring for people living with dementia.

Hartung et al. (2021) explored the experiences of new graduate nurses when providing care for patients with dementia in acute care environments. They found that the barriers to providing dementia care in acute care experienced by graduate nurses were similar to barriers experienced by all nurses, such as challenges with responsive behaviours, maintaining safety and providing psychosocial care. Facilitators identified were supportive colleagues and early exposure to dementia care.

Buenconsejo (2022) also explored new graduate nurse’s lived experiences in caring for hospitalized patients living with dementia. The themes identified in this study contributed to the understanding of the phenomenon discovered within the new graduate nurse’s lived experiences. She identified that graduates feel an obligation to protect patients living with dementia from actions that could harm them. Caring for people living with dementia was seen as complex, which affirmed the challenges of the new graduate nurses as they provide care amidst a highly stressful work environment. Graduate nurses strove to create meaningful connections with patients and believed these connections could cultivate harmony of body, mind, and spirit. The challenges new graduate nurses face when transitioning to their new roles were discussed as “nurse’s positive adaptation,” “role transition,” “feeling inadequate” and “experiencing personal distress”.

## Discussion

This scoping review aimed to review the available literature on graduate nurses caring for people living with dementia. This review has identified three themes from the literature: preparation to care for people living with dementia; experience of caring of people living with dementia; and impact of dementia care training.

Preparation of graduate nurses to care for people living with dementia is essential as graduate nurses contribute a significant portion of the nursing workforce across the globe (American Association of Colleges of Nursing, 2024, April; Department of Jobs and Small Business, 2018; OECD, 2021). Adequate dementia care training should be provided to nurses during both pre and post-registration programmes to enable them to provide quality care to their people living with dementia (Fessey, 2007). The undergraduate nursing programme should include an older people-friendly curriculum with adequate focus on gerontological nursing (Garbrah et al., 2017). It is expected that nursing students will develop skills required to practice as an independent registered nurse (RN) during their undergraduate training programme (Graf et al., 2020). But studies have raised concerns over the inadequate dementia care training offered by universities/colleges to undergraduate nursing students globally. Poor knowledge, confidence and moderate attitudes of dementia were found among nursing students in England (Baillie et al., 2015), India (Poreddi et al., 2015), USA (Kimzey et al., 2016), Turkey (Korkmaz Aslan et al., 2023), Indonesia (Sunaryo et al., 2020), Jordan (Aljezawi et al., 2022) and Australia (Eccleston et al., 2015).

Factors including educational preparedness, the gap between learning and practice, and the working environment can affect the clinical performance of newly graduated registered nurses (Henderson et al., 2016). Newly graduated nurses who do not experience adequate dementia care training during the university study period or who do not have any previous experience in caring for patients with dementia are at risk of sub optimal dementia care knowledge (Baillie et al., 2015; Duggan et al., 2013; Kimzey et al., 2016). Inadequate dementia care knowledge of graduate nurses may affect confidence, and the quality of care provided to people living with dementia (Theisen & Sandau, 2013). This lack of knowledge may also affect job satisfaction and increase the risk of burn out in the workplace (Kavanagh & Szweda, 2017).

Graduate nurses experience a transition whilst changing their role from student to independent nurse which often involves stress, shock and a steep learning curve (Duchscher, 2012; McCalla-Graham & De Gagne, 2015). Inadequate preparation to be an independent nurse (Flinkman & Salanterä, 2015), the busy hospital environment (McCalla-Graham & De Gagne, 2015) and a mismatching of their perceptions of nursing with the reality experience can be the main reasons for newly graduated nurses to feel stressed and shocked (Duchscher, 2012). Caring for people living with dementia, for which they have not been adequately prepared further increases the level of transition shock (McCalla-Graham & De Gagne, 2015). Some nurses caring for people living with dementia in the hospital settings find dementia care as challenging and burdensome (Byers & France, 2008). This negative attitude of nurses can affect the quality of care provided to the people living with dementia. It can also lead to frustration, reduced psychological well-being, feelings of powerlessness and eventually to job dissatisfaction (Byers & France, 2008; Griffiths et al., 2014). Nurses caring for people living with dementia in the hospital setting frequently cope with challenging issues such as calling out, wandering, resisting, aggression and agitation (Gladman et al., 2012). Constant exposure to challenging behaviours of people living with dementia often results in stress, exhaustion and frustration among the nurses caring for these people (Griffiths et al., 2014). Thus, the negative attitude and inadequate knowledge of managing people living with dementia can result in stress and burnout of nurses (Byers & France, 2008).

Dementia care education can impact the care of patients and the satisfaction of graduate nurses (Newman, 2020; Sorrell & Szweda, 2015). The Australian Institute of Health and Welfare (AIHW) (2018) has identified dementia care training of nursing staff as a key factor in improving the quality of care and outcome of the patients with dementia when admitted to hospitals. Adequate dementia care knowledge of nurses has a direct effect on improving the outcomes of patients with dementia and can reduce adverse triggers (Borbasi et al., 2006). Nurses who are competent in dementia care have a critical role in the timely diagnosis of medical conditions, implementation of appropriate interventions and maintaining the quality of care provided to patients with the condition (Sullivan & O’Conor, 2001). A study conducted by Wang et al. (2017) in China has identified that dementia care training has improved nurse’s dementia knowledge, attitudes, and intentions to achieve early detection of dementia. Piirainen et al. (2023) who undertook a study in nursing homes in Finland found that nursing staff training can decrease the prevalence of challenging behaviour among older people with dementia and enhance nurse’s competence in nursing. Dementia care knowledge and skills are deemed as essential to deliver high quality of care for people living with dementia admitted to hospital (Chater & Hughes, 2013). Nurses acquiring a high level of dementia care knowledge and positive attitude have demonstrated enhanced nursing care, especially compassion, emotional and cognitive empathy for people living with dementia, resulting in the provision of quality care (Evripidou et al., 2019). A study conducted in Taiwan found that the nurses who had attended dementia care training course were found to have better dementia care knowledge, which would improve nursing attitude and self-efficacy (Lin et al., 2024).

Smythe et al. (2020) undertook a study in UK showed that dementia care training provided to the nurses had positive impact in improving the quality of care provided and reducing the risk of burn out. A significant part of the WHO global plan is to train health care providers to care for people living with dementia to meet their special needs (WHO, 2012). To accomplish this target, the WHO (2012) recommends the development of dementia care training programmes for all health care professionals to encourage them to adopt positive attitude towards people living with dementia. Promoting dementia care training can improve healthcare requirements, thereby enhancing the quality of life of people living with dementia and the people caring for them.

## Recommendation

There were no studies to assess the dementia care knowledge and skills of graduate nurses, providing significant scope for research to enhance the dementia care knowledge and skills of graduate nurses. This area of research will directly help to enhance the quality of care provided to people living with dementia and may reduce the staff turnover. Research should also assess the undergraduate nursing curriculum from a variety of universities around the globe to check whether sufficient focus is given to dementia care knowledge during the Bachelor of Nursing programme. This focus on dementia care in the undergraduate curriculum may encourage universities to implement strategies to ensure that the nursing students will be competent to take care of people living with dementia with dementia when they start working as independent registered nurses.

## Conclusion

This scoping review has found that there is limited research literature about graduate nurses caring for people living with dementia. Adequate dementia care knowledge of graduate nurses is essential for the wellbeing of the patients as well as the graduate nurses caring for them. Little is known about the dementia care knowledge and confidence of graduate nurses caring for people living with dementia. There are opportunities for future research to explore the existing dementia care knowledge, confidence, experience and support needs of graduate nurses providing dementia care. Studies on the impact of dementia care training for graduate nurses are also of importance. The findings of this scoping review will add to a growing evidence base which will be strengthened by further robust studies exploring the support needed for graduate nurses to enhance their knowledge and confidence, thereby enhancing the quality of care provided to the people living with dementia in the hospital and community settings.
